# Secondary psychiatric care patients’ experiences of internet CBT for insomnia – a qualitative study

**DOI:** 10.1186/s40359-022-00943-0

**Published:** 2022-10-27

**Authors:** Frej Nicolaisen Sidén LP, Fredrik Spak

**Affiliations:** 1 Substitutionsmottagning dagvård & Olskroken, Sahlgrenska University Hospital, Olskroksgatan 30 Plan 3, 413 45 Gothenburg, Sweden; 2 Sahlgrenska University Hospital, Journalvägen 5, 416 50 Gothenburg, Sweden

**Keywords:** Insomnia, Sleep, iCBT-I, CBT, Internet, Online, Comorbid, Psychiatric, Secondary care, Motivation

## Abstract

**Background:**

Insomnia is very common, especially among psychiatric patients in secondary care. It is experienced as a 24 h problem affecting several domains of life. Cognitive behavioural therapy for insomnia (CBT-I) is widely regarded as the first-line treatment and often improves comorbid disorders. Despite this, many patients are not offered CBT-I. Internet based CBT for insomnia (iCBT-I) is just as effective as face-to-face treatments and could considerably increase availability. However, it is unclear whether iCBT-I is suitable for patients with more severe and comorbid psychiatric illnesses in secondary care.

**Methods:**

Eleven Swedish participants (24–68 years old) in outpatient secondary psychiatric care who underwent iCBT-I treatment were interviewed. The semi-structured interviews were analysed using content analysis. The purpose was to map their experiences, motivation and suggestions for improvement.

**Results:**

Prior to the treatment, most participants were highly motivated to take the opportunity to address their insomnia. The treatment was perceived as well-structured and interesting. The most difficult aspect was counteracting the fatigue. It was also hard to continue the treatment when faced with major life events. During this phase, contact with the therapist motivated them to continue the treatment. Several desired more face-to-face meetings. It was also motivating to gain insights into and a sense of control over sleep. Several described better sleep, improved daily routines, a more predictable everyday life and increased energy. Daytime well-being was improved in some, partly because they had more energy but also because they filled their days with more activities.

**Conclusion:**

The treatment has the potential to be very useful in secondary psychiatric care where insomnia is common and affects comorbid disorders. Psychiatric patients might have more difficulties continuing with iCBT-I treatment, but those who manage to complete the program have a good chance of obtaining benefit. Extensive psychological groundwork early in treatment is likely to pay off later when motivation is needed. Additional social support and other adjustments may also enhance treatment outcomes. Participants’ stories are particularly valuable, as therapists and treatment developers receive less feedback from patients in internet-based treatments compared to face-to-face treatments.

**Supplementary Information:**

The online version contains supplementary material available at 10.1186/s40359-022-00943-0.

## Background

In the fifth edition of the Diagnostic and Statistical Manual for mental disorders, DSM 5, insomnia is defined as ”a predominant complaint of dissatisfaction with sleep quantity or quality” pertaining to initiating or maintaining sleep or early-morning awakening causing “clinically significant distress or impairment in social, occupational, educational, academic, behavioural, or other important areas of functioning” [1, p. 362]. It is experienced as a 24 h problem that affects several domains of life [5].

Insomnia is a common problem with a point prevalence of 6–15% in the general population [26]. It may also be caused by various mental or medical disorders. Irrespective of whether insomnia is caused by other disorders, it is a problem that requires independent clinical attention [9, 20].

A qualitative review of patients with insomnia found that there was often a mismatch between the patients’ perspective and that of healthcare professionals regarding the nature and treatment of the problems. Patients frequently experienced that healthcare professionals focused too much on medications and sleep hygiene [5].

Cognitive behaviour therapy for insomnia (CBT-I) is a treatment that in addition to sleep hygiene includes stimulus control, sleep restriction, cognitive therapy and relaxation. It is regarded as the first-line treatment for insomnia by many organisations and guideline panels [24, 25, 27, 28]. CBT-I has a similar effect to pharmacological treatment in short-term treatment and has been found to be more effective in the long term [14, 28]. CBT-I has also been shown to be effective in improving sleep for patients with psychiatric comorbidity such as anxiety and depression [9, 17]. Despite this, many patients are not offered CBT-I.

Delivering CBT-I in internet format (iCBT-I) could considerably increase its availability. Such a format is more time-efficient for the therapist (10–15 min/week) and patients can take part in the treatment at their own pace, irrespective of their geographical location and time of day.

The results from meta-analyses of iCBT-I provide support for improved sleep in both short and long-term follow-ups [12, 13]. In previous studies participants have usually been recruited from the general population via the media [22]. The studies of iCBT-I conducted in psychiatric populations also show good results with reduced comorbid anxiety and depression [4, 13]. Most of these studies have focused on insomnia patients with comorbid depression. In a review of adherence to CBT-I, mainly face-to-face CBT-I, some signs were found that comorbid depression and anxiety may predict attrition in CBT-I but it was concluded that there is a knowledge gap regarding comorbidity and adherence [21]. There have also been indications that therapist support is particularly important in written self-help CBT for insomnia compared to other CBT treatments [15], although it is unclear to what degree this also applies to internet-based treatments and to patients with comorbidities.

One issue raised in a qualitative study of iCBT-I is that the need for therapist support may be greater in patients with comorbid depression [2]. Other findings from qualitative studies of iCBT-I that included patients with psychiatric comorbidity indicated that the treatment benefitted participants who were goal-directed, liked graphic interpretations and were intrinsically motivated to improve their overall health [11]. Acceptance of problems related to insomnia as well as of negative emotions and cognitions also seemed to facilitate a good outcome among patients with comorbid depression [8]. Difficulties reported in a qualitative iCBT-I study of patients with comorbid substance use disorder were that the treatment was regarded as time-consuming as many had competing demands, that it lacked individualization, that it was boring and that the format was similar to schoolwork [18].

More qualitative studies of iCBT have been conducted for other types of psychiatric disorder. A meta-synthesis of 23 qualitative studies on the acceptability and usability of digital health interventions (DHI) for depression, anxiety and somatoform disorders was recently published [29]. Three major themes were found: initial motivation and approaches to DHI, personalization of treatment and the value of receiving personal support. Implications related to the themes were proposed. One was to address expectations prior to the start of DHI to help manage any misconceptions and early barriers in addition to assessing preferences pertaining to autonomy and support. Another was to personalize the interventions in the sense of communicating about the purpose, having a good user interface or using feedback and reminders, making it easier for users to commit to and engage with the intervention. The third proposed implication was to make rapid human support available, particularly to users with a low level of engagement or difficulties.

In another qualitative meta-synthesis of user experience of computer therapy for depression and anxiety there was also a focus on personalization and support [7]. Users in the studies expressed a need to experience a sense of “self” and identity in the programmes. Other patterns found were pros and cons regarding the level of independence/support and contact/privacy in relation to others.

A review of 19 qualitative studies examined the recruitment and engagement process in a broader spectrum of digital health interventions [23]. One of the recommendations was to incorporate social contact with care providers or peers with similar health issues to enhance engagement and enrolment, so that users can quickly and easily access the support they need. They also highlighted the need for DHI to “make sense” to the users during the pre-treatment recruitment.

### Research aim

To evaluate the outcome of an internet-based CBT treatment for insomnia in psychiatric secondary care using interviews with patients who had undergone such treatment.

### Research questions

- What are the participants’ expectations before undergoing iCBT-I treatment?
- What are the participants’ experiences of the treatment?
- How do the participants think the treatment can be improved?

## Method

### Design and participants

Eleven participants were interviewed about their experiences of iCBT-I treatment. The treatment contained best practice CBT-I components [24]. It was specially adjusted to psychiatric patients by means of more videos, briefer information and focus on the core components, i.e., sleep restriction and stimulus control. These components consist of behavioural changes intended to create sleep pressure and stabilise circadian rhythms. The other CBT-I components included were information on sleep, goal formulation, sleep hygiene, relaxation exercises to minimise pre-sleep arousal, daytime behavioural activation to counteract fatigue, writing exercises and behavioural experiments aimed at altering sleep-related misconceptions and worry, and finally insomnia relapse prevention. The patients received the same standardized treatment, but all exercises had an individualized application. They could take part in the eight-module treatment at their own pace but were encouraged to finish one module per week. Sleep patterns and progress were tracked daily (sleep diary) and weekly (Insomnia Severity Index and PHQ-9). The results were visible for both patients and therapists during the entire treatment period. The participants received therapist support through written messages at least once a week and phone calls if they had been inactive for a couple of weeks. The six therapists involved in the treatment were licensed psychologists or CBT-therapists, who had received at least one day of iCBT-I treatment training and had access to a treatment manual and supervision.

The treatment and interviews were carried out in clinical practice settings in Gothenburg, Sweden, during spring and summer 2019. Gothenburg is a city with a population of 600,000, making it the second largest city in Sweden. Three adult outpatient secondary care psychiatric clinics at Sahlgrenska University Hospital were included; two general psychiatric clinics and one opioid substitution treatment clinic. More information on the implementation and context of this particular iCBT-I treatment is provided in Banck & Bernhardsson [6].

In the present study, patients with insomnia were asked to participate after initial screening. Participants were included if they had mild to severe insomnia (> 11 on Insomnia Severity Index) and no other sleep-wake disorders (e.g., sleep apnoea, narcolepsy, hypersomnia). The inclusion criteria also stated that patients needed to be technically equipped and able to use a computer, have sufficient knowledge of the Swedish language, not be in an acute condition and not on such heavy medication that their cognitive abilities were impaired. In addition to insomnia, many of the participants had other diagnoses such as anxiety disorders, depression, neuropsychiatric disorders, personality disorders and medical co-morbidities. Participants with opioid substitution treatment had maintenance therapy and no other substance use disorder. Patients with bipolar disorder, epilepsy (seizures in the past year) and psychosis (symptoms in the past year) were excluded because such patients require special treatment to prevent relapses triggered by sleep restriction.

The participants were recruited by many different health professionals at the three clinics as part of their everyday practice. The selection was based on the need for treatment rather than the aim of the present study. The selection criteria are described above. All patients who started the treatment were invited to participate in interviews. Eleven participants were interviewed, eight women and three men, aged 24–68 years (mean: 39 years). In planning the study, we envisaged 10–25 participants to be an appropriate number. The final number was in the lower range because saturation was reached after 8–9 participants. Any additional participants would most likely have made some contribution, but after eleven interviews the subject was broadened and explored to a sufficient degree. We also prioritised adhering to the time schedule by not continuing the recruitment process. One participant was excluded from the study because she suffered from hypersomnia. Half of the participants completed all eight modules, which provided an adequate degree of variation. For more information on the participants, see Table 1. All interviews were semi-structured and conducted over the telephone. The interviews lasted between 17 and 32 min (mean: 25 min).

**Table 1:** Participants

Participants	Male/Female	Completed modules (8 models in total)
1	F	8
2	F	6
3	F	8
4	F	8
5	F	3
6	F	8
7	F	7
8	F	8
9	M	2
10	M	8
11	M	6

### Interviews and analysis

The study had a pragmatic worldview with focus on how the digital intervention could make life easier for people with insomnia. The pragmatic approach is in line with the studied CBT intervention.

The topics covered in the interviews were motivation prior to the treatment, experiences of the treatment, evaluation of the therapy as well as the internet format and how comorbid psychiatric problems interacted with the treatment (see the interview guide in the appendix). The topics remained the same throughout the study and were considered to have face validity in relation to the research aim. The question on motivation is commonly used in motivational interviewing to explore motivation and ambivalence [30]. Expectations, motivation and identification have been highlighted in previous research on CBT-I. All questions required reflection but could also be answered by participants with cognitive impairments. The follow-up questions were open and non-directive in order to give the impression of a natural conversation.

The interviews, transcripts and coding were performed by FNS, a clinical psychologist with education in and experience of CBT-I treatment as well as a basic university education in research methodology. FNS had no previous relationship with any of the participants but was a colleague of three of the six therapists. FS had no previous relationship with any person in the study.

A content analysis was conducted [19]. This method of analysing qualitative data is suitable for the aim of the present study, which was to capture different aspects of the user experience and the utility of the iCBT-I intervention. The de-identified interviews were transcribed verbatim. The de-identification did not affect the meaning of the data. When the researchers had familiarized themselves with the content of the transcribed material, the data were condensed, and the manifest meaning coded. Threads of meaning were then identified and grouped. The research aims guided decisions regarding what to look for and how to differentiate between groups of content. The summarised categories and subcategories were first grouped, after which underlying themes and subthemes were searched for. The interpretation was inductive and empathetic in that it was open-ended and exploratory with the aim of understanding the participants’ experiences and perspectives. The interpretation also had some deductive and critical elements, as CBT-theory was applied and attempts were made to understand the implicit and latent meaning. In the analysis process there were no attempts to determine whether the treatment worked. Instead, the focus was on whether the treatment was experienced as feasible and on gathering nuanced and rich information about circumstances that facilitated or complicated it. Saturation was considered to be achieved after eleven interviews, as the last three interviews fitted into the previous categories and themes and did not provide any new information. The entire material was then related to the context and compared with the literature to enable further reflection. CBT theory [10, 16] was applied to understand the therapeutic process. One fifth of the data were recoded with a more empathetic interpretation to ensure that the themes captured the latent emotional meaning. We found no major discrepancy in the comparison between the codes that evolved in that process and the themes. The authors discussed the coding framework and themes at regular intervals. Both researchers were engaged throughout the process and discussed all important steps taken. Awareness of and supervision to prevent distortions and misconceptions of the material were prioritized. The themes were discussed with a third PhD researcher (AJ) who has vast experience of qualitative research. The four themes were finally divided into 14 subthemes, which are presented in Table 2.

## Results

**Table 2:** Themes and Subthemes

Themes	Subthemes	Examples of quotations
Finding my place in the treatment	Engaging with computerized treatment	“I mean it was all very clear.” (3)
	Relating to the treatment content	“Some of it I could definitely relate to, but not all.” (9)
	Therapist support makes a difference	“And maybe because of her I started to tackle things (laughter) I don’t know. But sometimes you need more push.” (7)
	Avoiding and adjusting the difficult parts	“So the sleep restriction in particular I felt wasn’t really working for me. But I think it works very well for other people.” (6)
	Adhering to the overall treatment plan	“It probably would have worked better if I hadn’t had so much else on my mind.” (9)
Dealing with sleep problems	Experiences of sleep problems	“I think this is very big, that you can sleep. That says it all I think.” (7)
	Gaining new perspectives on sleep	“The body works after all, it feels important to know those things because then it’s like I’m giving it another chance.” (2)
	Difficulties counteracting fatigue	“It is not easy when the whole body is dog-tired but you still cannot go to sleep.“ (1)
	Starting new habits and routines	“It has done me good - structure maybe, that I got some sort of schedule to go by.“ (8)
Striving towards what might be a possible solution	Reasons for trying	“So you don’t lose it and forget about it and so on, I guess that might be the only thing. But otherwise it was all really positive.” (10)
	Coping with self-responsibility	“If you have an appointment booked at a clinic, you know about it and plan for it and go there. Here it is more free. But it suited me very well.” (7)
Building up energy for the day	Emotional changes	“It made me calmer knowing that I sleep better, I get rest. Gave me confidence in the other parts as well.” (11)
	Getting on with everyday life	“I ended up having energy to do stuff during the day.” (2)
	Increased energy in relating to others	“Before I was so tired that I did not hear, I heard nothing, I saw the people, that people were talking but I did not hear their voices sometimes … Today I can manage a little more.” (7)

### Finding my place in the treatment

The patients could log in quite easily and get started with the treatment. The first informative parts about sleep aroused curiosity. After a while things became somewhat more difficult. Adhering to the treatment plan was not always easy and at that point having therapist support available was beneficial. As almost the entire treatment was tough, many asked for more support, while others wanted to avoid or adjust certain parts to make it more manageable.

**Engaging with computerized treatment.** The internet format was perceived as structured, clear, flexible and with a good flow. The participants appreciated not having to travel back and forth to meetings and being able to carry out the treatment at any time – even at night. However, the sleep diary could sometimes be complicated, unclear and lack certain aspects. Another issue raised was the need for a more cheerful web design and a more positive atmosphere in the videos. The treatment as such was not experienced as technically complicated.

Participants who had impaired cognition read the material several times. One participant with dyslexia asked for the text to be read aloud.

**Relating to the treatment content.** Most participants could relate to the treatment content. The participants repeatedly expressed that the information about sleep problems was interesting, instructive and clear. Although most of it was already familiar to them, it aroused their curiosity and provided insight.

The part that dealt with worries was often mentioned by the participants and some reported feeling confirmed by their worries being raised and addressed. Others thought that there was too much information about worries – “it is pointless getting excited about it!“ (1) and asked for more examples of helpful thoughts when lying awake.

**Therapist support makes a difference.** The therapists sent messages to their patients via the treatment platform. They also contacted the participants by phone if they had been inactive for a couple of weeks, which motivated them to continue changing their habits. The participants were mostly satisfied with the support they received from the therapists but would have liked even more support. They requested face-to-face meetings to obtain feedback, motivation, follow-up, the opportunity to ask more questions or because it becomes “something else when you also meet face-to-face at some point” (8).

**Avoiding and adjusting the difficult parts.** Some skipped the sleep restriction section, perhaps the most difficult but at the same time one of the most effective parts of the treatment. Participants often changed the pace or took breaks during this difficult section, which could make the treatment feel less demanding and more suited to their individual needs. Skipping sections was also seen as avoidance associated with negative emotions.

**Adhering to the overall treatment plan.** There was a wide variation in treatment persistence. Some dropped out after a couple of modules, others remained for half of the treatment, while most completed the main part of it. The general impression of the treatment ranged from “I was probably not the right person for this particular treatment” (5) or it “went so-so” (1) to “it was one of the best things I’ve done for myself in my life” (7). Most were satisfied with the treatment.

A daily link to the treatment consisted of writing the sleep diary, which sometimes lacked continuity. One participant “logged in on Friday night and filled in for the whole week”, which made it confusing and “difficult to maintain after a while” (2).

Those who dropped out early usually said other major events got in the way, e.g., separation from their partner, major surgery or starting a new job after unemployment.

One participant found it difficult that a large part of her treatment occurred during her holidays. She thought it would be “easier to do it when you have some kind of everyday life with routines” (8).

Of the participants who completed the treatment, several continued to work with it after the eight weeks had ended by printing the material, logging in and starting again or just continuing to apply the lessons in their everyday life.

### Dealing with sleep problems

Having had insomnia for a long time, it was time to make some change. Doing so was tough, as some techniques required a thorough change. Receiving clear instructions and routines about the things they had been struggling with for a long time was one of the most helpful parts of the treatment. Those who persisted were rewarded with new insights.

**Experiences of sleep problems.** Many described having had insomnia for a long time. Some related it to other problems such as pain, brain fatigue and anxiety. One participant with anxiety found it “very difficult to find peace and fall asleep, because then the thoughts come”. She was “afraid to fall asleep” (5), which became a barrier to completing the treatment. Those who were helped by the treatment described that they fell asleep faster, slept heavier and longer, had fewer awakenings and were more rested during the day. Some mentioned that they became less anxious and had a “more relaxed attitude” (8) when unable to sleep at night but also during the day.

**Gaining new perspectives on sleep.** The treatment provided the participants with insights and perspectives on their sleep. Even those who finished the treatment early described that their attitude changed after reading the first module that dealt with myths about sleep. Those who completed the treatment described major changes in perspective due to both the information provided and their own experiences.

Participants described having a clearer picture of how they actually slept thanks to the sleep diary and diagrams. One insight was that even if they had slept poorly, they were nevertheless able to cope with the following day, which had a calming effect.

**Difficulties counteracting fatigue.** The treatment involved tasks in which the participants were encouraged to counteract fatigue – getting out of bed despite extreme tiredness, trying to stay awake to build up sleep pressure and not sleeping during the day. These experiences were difficult and often described as a burden. It was not easy getting out of bed “when the whole body is dog-tired but you still cannot go to sleep” (1). The participants could relate their tiredness to aspects such as hormonal changes and brain fatigue. One participant asked for more suggestions on activities she could perform to stay awake. Although it was hard, in retrospect some saw it as part of the process. It was “difficult at first not to fall asleep in the daytime” (2) and “a completely different routine than what you are used to, so it’s no wonder you find it difficult” (2).

**Starting new habits and routines.** Participants who noticed progress in the treatment mentioned that they knew what to do if they were unable to sleep, which they considered one of the most helpful parts of the treatment, as it provided a sense of control. They gave many examples of “if X happens, I’ll do Y” or talked more generally about having “found routines”. These routines made the evenings, nights and mornings more predictable. One participant described routines as creating “some sort of schedule to go by” (8). Another stated that she “didn’t see certain things before” but now she both sees and “fixes it as best I can” (7). “When you should do something, you think back and like yeah, that is what she said” (1). One participant expressed that it was “stabilizing” to be instructed to “do these things, think about this” (11).

It was not always easy to implement the new habits. Some days they felt incapable of trying. They could be stressed, feel listless and lose their motivation. This alternated with days when they tested to see if the treatment produced any results. One participant only read the texts for a month before reluctantly making some attempts to implement the changes of habit she had read about. She developed more motivation when she noticed that the changes worked – “Honestly, at first everything seemed very ridiculous. You just can’t believe it – you force yourself to do certain things” (7).

### Striving towards what might be a possible solution

When the participants were invited to the treatment, they had varied expectations. Most perceived it as a very good idea but were more or less discouraged by uncertainties about whether they would be able to to adhere to it. The self-responsibility that came with the internet format could be both a burden and a basis for freedom.

**Reasons for trying.** The participants were generally highly motivated before starting the treatment. The motivation was mainly derived from the fact that they wanted to overcome their sleep problems. Some mentioned that they wanted to cease taking their sleep medication and “do something yourself” (8). Another common source of motivation was their impression that those who provided the information about the treatment firmly believed in it themselves.

The most common cause of doubt was whether they would be able to complete the treatment. They recalled previous experiences of not accomplishing things due to having failed or forgotten.

One person had no motivation whatsoever before the treatment because she had previously tested several other methods and “didn’t think anything could help me” (7) and “some things don’t work at first or it’s so ridiculous” (7). “It was my psychiatrist or psychologist who wanted me to try. And I thought OK, I’ll give it a try” (7). Another source of ambivalence was mentioned by a participant who had become accustomed to her chronic insomnia and did not want to upset the current status quo. After thinking about it, she decided to take part in the treatment because “I can only learn more that might be useful to me” (3).

When the participants had been inactive for a while the therapists contacted them, which the participants described as motivating. Another common source of motivation was experiencing progress in everyday life and following one’s sleep diagrams.

**Coping with self-responsibility.** Compared to face-to-face treatment, internet treatment was generally perceived as requiring a higher degree of self-responsibility. Some thought it suited them well, while others considered it too difficult. The most demanding part was not the amount of text and videos involved but changing everyday habits. The autonomy involved in using the internet format was experienced by some as a burden.

For others the internet format was perceived as less demanding compared to face-to-face treatment. One participant who was not very motivated before the treatment agreed to take part because the internet format made it easier, as he was free to do it at his own pace, thus creating less stress.

### Building up energy for the day

Improved sleep generated energy and gave a sense of control. The participants could also gain energy by activating themselves to become more energetic. A more stable sleep pattern made it easier to plan activities and find routines. Not all improved and those who did not could become disappointed and self-critical, at least for a while.

**Emotional changes.** Those who reported enhanced overall mental health mainly explained that it was due to having more energy because of improved sleep. It also seemed to be related to the new routines and activities during the day. Some also mentioned that they had become “calmer” as well as “feeling safer” (11), with an increased belief that “the body takes care of itself” (6) and now had “better self-confidence” by “daring to cope” (2).

There were also several who stated that better sleep did not affect their lives in general, “not much unfortunately, not much. No more than that I get to sleep” (4).

Participants who had major difficulties and those who dropped out early sometimes described increased negative emotions – “one clanks down pretty much on oneself” even though “there wasn’t much I needed to do really” (5). “As I said, I am very disappointed that I was not able to do it fully” (1). They pointed out, however, that the treatment did not have an overall negative impact on them, although their anxiety and depressed mood increased as a result of encountering various difficulties.

**Getting on with everyday life.** The days were sometimes filled with activities such as “meeting my friends” (2), “doing a workout“(1) or just changing into “ordinary clothes” (7). These were ways to get the day started, counteract fatigue and become more tired in the evening – “to stay awake you have to activate yourself. And then you automatically try to go out for a walk or something just to do something” (1).

Improved sleep sometimes provided more energy and strength in the daytime. The participants described being “more alert” (8), able to “work better during the day” (2), “withstand a little more” (7) as well as feeling “more alive today, that my life has some quality” (7).

As participants developed more consistent sleep patterns they found it easier to plan their days **–** “there’s more order in my life. I can plan the days now, like what I should do. Before I didn’t know how I felt. I didn’t know if I could manage it the next day” (7).

Not all participants managed to activate themselves. One felt that the hardest part was “to activate yourself during the day, I haven’t got to that yet” (4).

**Increased energy in relating to others.** Those who mentioned that relationships were affected by the treatment described positive changes. It was easier to relate to others and take initiatives when they had more energy.

## Discussion

Prior to the treatment the participants’ motivation was usually high, as they considered it a good opportunity to deal with their insomnia. Doubts mainly arose due to previous experiences of being unable to complete similar programs. Those who completed most of the treatment reported gaining new routines that gave insights and a sense of control over sleep. These experiences motivated them to proceed. Phone calls and messages from the therapist were also motivating. The internet format was regarded as well-structured and easily accessible, although generally perceived as requiring a higher degree of personal responsibility than face-to-face treatment. Some stated that this suited them well, while others requested more therapist support. The hardest parts were counteracting the fatigue and engaging in sleep restriction. Many participants were reluctant to continue the difficult tasks, and dropouts were especially frequent during sleep restriction. Other causes of dropout were life events, e.g., major surgery and separation. Dropout could cause disappointment and self-criticism but was not perceived to have any negative long-term impact. Many of those who completed most of the treatment described improved sleep at night and increased energy during the day, making it easier to socialize and be more active, which enhanced their quality of life.

### Comparison with existing literature

Much of what emerged from previous studies was also present in the participants’ stories in the present study. Before the treatment began, they had different expectations. Many patients started the treatment with some uncertainty about what to expect, whereas others had fairly high motivation. Although some found it difficult to believe in the effectiveness of the treatment, the most discouraging aspects were being reminded of past failures and lacking confidence in one’s ability to adhere to the treatment. There were also concerns about the internet format. This stage, when the patient get in contact with the treatment and gets started, has been highlighted as important in previous studies [23]. It has also been seen as important that early expectations, misunderstandings and barriers are addressed [29], in addition to the patient feeling that this is a treatment for me that has a place in my everyday life [7]. For psychiatric patients, everyday life can take many different forms, with higher rates of unemployment, physical illness, relational problems, temporary crises and other difficulties, compared to the general population. This lack of routines can make adherence to the treatment more difficult.

Many also encountered major problems a few weeks into the treatment when sleep restriction began. This important part of the treatment has previously been identified as difficult in that it is often perceived as counter-intuitive and demanding [21]. Participants in the present study found therapist support motivating during this stage. They struggled to adhere to the new habits and had questions about whether they were on the right track. More support was requested and some of them suggested group discussions to share experiences and gain motivation and feedback. Previous studies have highlighted the need for rapid support [23, 29]. In the present study, the ease of access to support was mentioned as positive by several participants, while the lack of rapid support was mentioned by one as negative. Capturing the motivation before it weakens might be important, as the internet format may increase the risk of procrastination and avoidance in some patients.

Something that may have been underestimated in previous research is the role of the therapist as ‘supervisor’. A significant part of the motivation to continue despite difficulties may derive from the negative reinforcement of knowing that the therapist will be disappointed if the assignment has not been accomplished. Such negative reinforcement needs to be combined with a great deal of positive reinforcement and reminders of how the tasks relate to the patient’s goals. There is also a risk of the therapist lowering the expectations on the patient too much if the latter runs into difficulties and exhibits emotional avoidance. This risk exists in all therapy and there is reason to believe that the standardization of internet treatments to some extent protects against such “therapeutic drift”. Nevertheless, these are further reasons to be careful with the pre-treatment clarification of purpose, expectations and barriers, thus allowing patients to find a ‘self’ in the treatment, discussing preferences regarding the level of support, medium of communication and other aspects of the therapeutic frame – especially in psychiatric care. Ideally, the support provided should enhance the patient’s competence and behaviour control, as these are important for motivation [31, 32] and were common pre-treatment barriers in the present study.

The ePsychiatry unit at Sahlgrenska University hospital [6], where the internet treatment was designed, changed its recommendations after this study was completed. They recommended that therapists should make a follow-up face-to-face call (video call or in real life) after about four weeks during the sleep restriction phase, a time when dropouts are common. Another change in recommendations was to exclude patients with severe depression (defined as 22 or more points on the Patient Health Questionnaire-9, PHQ-9). They saw large improvements among patients with low or moderate depression, but no improvement and high drop-out rates (2 out of 3) among patients with severe depression.

However, providing treatment with a good deal of support makes it more resource-intensive, less scalable and thus available to fewer patients. This is a dilemma – a trade-off between providing poor-quality treatment to many or high-quality treatment to a few. In specialized psychiatric care it is probably necessary to have a high level of personal support, even if it means that fewer patients benefit. The patients need to feel that there is someone providing rapid support when things are difficult, who will get back to them when the difficult tasks are not being accomplished. To make it more resource-efficient, parts of the support can be automated, e.g., through an accessible interface, introductory and motivational interviewing modules preceding the actual treatment, automatic reminders and automatic rewards for completed home assignments, clear feedback on progress or through moderated discussion forums or group meetings with other patients attending the treatment.

As insomnia is a relatively non-stigmatised form of mental illness, support does not have to come at the cost of reduced privacy. In the present study no participant mentioned stigmatization or that the written format made it easier to express their thoughts and feelings, which has been reported in previous studies on iCBT [7, 29]. This is probably because the treatment addresses common problems with everyday routines rather than “hidden” emotional problems.

### Strengths and limitations

One strength of the study is that it was conducted in an everyday clinical context at three different sites. As the treatment is new, it has been carefully evaluated within the organization by many different professional categories.

Even though the selection of participants was based on the need for treatment rather than the aim of the present study, it is possible that the study influenced the motivation of some patients or professionals.

There was probably a selection bias in that those who could benefit from the treatment were more willing to be interviewed. A third of the patients who were contacted did not want to be interviewed. Some mentioned that they had dropped out early and did not think they could contribute much to the study. The sample had a greater proportion of participants who completed the treatment, and thus fewer participants who experienced failures were interviewed.

Eight women and three men were interviewed. The reason for this imbalance is not known, but selection bias cannot be ruled out.

Another limitation was that the questions about motivation to initiate treatment were posed after the treatment had been completed. Some may have had difficulty remembering and the answers were probably coloured by their experiences of the treatment.

Only two persons were involved in the coding and analysis, which increases the risk of bias. A third researcher with vast experience of qualitative research also read and commented on the material.

### Possible implications

There are few qualitative studies of internet-based CBT treatment for insomnia in patients in a secondary psychiatric care setting. As insomnia is very common in this group, participants’ stories are valuable.

The study may increase understanding of the treatment from the patients’ perspective. Therapists in internet treatment receive less feedback from patients compared to those involved in face-to-face treatment. The study can be used in developing similar future treatment and guide therapists in recruiting patients and delivering treatment.

As was anticipated, patients’ progress in the programme varied. Psychiatric comorbidities sometimes hindered completion of the treatment. After screening and basic exclusion, the patients themselves are probably the best judges of whether the treatment is suitable for them. However, according to the ePsychiatry unit that developed this treatment, patients with severe comorbid depression have a low likelihood of success and can therefore be excluded.

Building a solid foundation from the beginning by addressing the patients’ goals, expectations, barriers and support preferences allows them to find their ‘self’ in the treatment and is likely to pay off at a later stage. Especially when motivation and self-efficacy to cope with sleep restriction and stimulus control become necessary.

Additional support and other individual adjustments may also enhance treatment outcomes.

## Conclusion

The treatment was useful for many of the participants interviewed in this study. It provided new routines that gave insights and a sense of control over sleep. Improved sleep at night often led to increased energy during the day, making it easier to interact with others and be more active. The treatment has the potential to increase the overall quality of life both at night and during the day. Sleep is an essential, but often neglected, component of many aspects of good psychiatric health. The internet format could considerably increase the availability of this treatment, which is highly relevant in secondary psychiatric care, where insomnia is common and affects comorbid disorders. Although this treatment can be very helpful, it was not without disadvantages. The important sleep restriction intervention was regarded as difficult by many. Dropouts were especially frequent during this part of the program. Other causes of dropout were significant life events, e.g., major surgery and separation. Psychiatric patients might have more difficulties continuing with iCBT-I treatment, but those who manage to proceed stand a good chance of deriving benefit. The higher degree of personal responsibility required has both benefits and disadvantages. Experiences of successful outcomes may improve self-efficacy. However, carefully preparing the patient for the treatment and providing easily accessible support during the process might be required to enhance motivation and make more patients willing to give it a try and to succeed.

In internet-based treatment, the therapists receive less feedback from patients compared to face-to-face treatment, making the participants’ stories in this study particularly valuable.

## Electronic supplementary material

Below is the link to the electronic supplementary material.

Supplementary Material 1

## Data Availability

The datasets generated and analysed are not publicly available due to content that could potentially identify the participants. They are, however, available from the corresponding author on reasonable request.

## Abbreviations

CBT: Cognitive Behavioural Therapy.
CBT-I: Cognitive Behavioural Therapy for Insomnia.
iCBT-I: Internet based Cognitive Behavioural Therapy for Insomnia.
DHI: Digital Health Intervention.
