# Supplementary material for: Secondary psychiatric care patients’ experiences of internet CBT for insomnia – a qualitative study
Source: BMC Psychol. 2022 Oct 27;10:237. doi: 10.1186/s40359-022-00943-0 (PMC9615376; doi:10.1186/s40359-022-00943-0)
Supplement: Supplementary file 1 — Supplementary Material 1 [file 40359_2022_943_MOESM1_ESM.docx]

**Appendix – Interview Guide**

- What was it like for you to participate in the treatment?
- What was it like to participate in the treatment via the internet?
- Did you complete the whole treatment or finish early?
  - (Can you please tell me what made you finish?)
- I will now ask a question where I would like you to answer with a number from 1 to 10. And for this question, please try to remember your attitude when you found out that you could participate in the treatment… How motivated were you at that time on a scale of 1 to 10, where 1 means that you were not at all motivated and a 10 means you were very motivated?
  - Why did you not choose a higher number?
  - Why did you not choose a lower number?
- Name five words that describe your experience of attending this treatment.
  - Please tell me more about what you mean with [words # 1, 2, 3, 4 and 5]
- The picture of insomnia that was communicated, how did it match your experience of insomnia?
- How have other psychological problems and difficulties in your life affected your experience of undergoing this treatment?
- How do you think this treatment has affected your other psychological problems and difficulties in life?
- Do you have any suggestions for improvements to those designing this and similar treatments?
  Can you come up with anything else that you think can be improved, or that those designing this and similar treatments should bear in mind?

I have no further questions, but is there anything you would like to add that has not been mentioned?
